# JAK/STAT Cytokine Signaling at the Crossroad of NK Cell Development and Maturation

**DOI:** 10.3389/fimmu.2019.02590

**Published:** 2019-11-12

**Authors:** Dagmar Gotthardt, Jana Trifinopoulos, Veronika Sexl, Eva Maria Putz

**Affiliations:** ^1^Department for Biomedical Sciences, Institute of Pharmacology and Toxicology, University of Veterinary Medicine Vienna, Vienna, Austria; ^2^St. Anna Children's Cancer Research Institute (CCRI), Vienna, Austria

**Keywords:** NK cell, ILC1, development, maturation, cytokine, JAK, STAT, SOCS

## Abstract

Natural Killer (NK) cells are cytotoxic lymphocytes of the innate immune system and play a critical role in anti-viral and anti-tumor responses. NK cells develop in the bone marrow from hematopoietic stem cells (HSCs) that differentiate through common lymphoid progenitors (CLPs) to NK lineage-restricted progenitors (NKPs). The orchestrated action of multiple cytokines is crucial for NK cell development and maturation. Many of these cytokines such as IL-2, IL-7, IL-12, IL-15, IL-21, IL-27, and interferons (IFNs) signal via the Janus Kinase / Signal Transducer and Activator of Transcription (JAK/STAT) pathway. We here review the current knowledge about these cytokines and the downstream signaling involved in the development and maturation of conventional NK cells and their close relatives, innate lymphoid cells type 1 (ILC1). We further discuss the role of suppressor of cytokine signaling (SOCS) proteins in NK cells and highlight their potential for therapeutic application.

## Introduction

Innate lymphoid cells (ILCs) comprise a variety of cell types with the morphological characteristics of lymphoid cells, but unlike adaptive immune cells, ILCs completely lack rearranged antigen receptors. In analogy to the classification of T cell subsets, ILCs can be sub-divided into three groups according to their dependence on distinct transcription factors and to their cytokine expression repertoire (1). Group 1 ILCs include two major members, conventional NK cells and ILC1s, both of which are characterized by the ability to produce T helper-1 (Th1) cell signature cytokines (e.g., interferon-gamma, IFN-γ) and by their functional and developmental dependence on the transcription factor T-BET. Group 2 cells (ILC2s) produce Th2 cell-type cytokines (e.g., IL-4, IL-5, IL-9, and IL-13) and depend on GATA3, whereas group 3 cells (ILC3s) are potent producers of IL-22 and/or IL-17A and are characterized by RORγt expression (1, 2).

NK cells account for 8–15% of circulating cells in the human blood or 2–6% in mouse blood, and are found throughout the body, in particular in lymphoid organs, lung, liver, uterus and gut (3). Similar to CD8^+^ cytotoxic T cells, NK cells are important in the defense against tumors and the spread of viral infections by producing pro-inflammatory cytokines such as IFN-γ and TNF-α. However, unlike T cells NK cells do not require prior sensitization and lack antigen-specificity allowing them to patrol and eliminate a broad range of altered and transformed cells. To do so, the activity of NK cells is controlled by a delicate balance of inhibitory and activating receptors, which interact with surface ligands and either prevent or trigger the lysis of a target cell (4, 5). Whereas, NK cells recirculate via blood and lymph vessels and have a license to kill, ILC1s are mostly tissue-resident and show low cytotoxic potential. Besides their common feature of being highly efficient IFN-γ producers, NK cells and ILC1s share many surface markers as well as transcription factors that complicates their discrimination especially under conditions of inflammation or in cancer (6, 7).

In humans, the identification of specific markers for ILC1s remains challenging (6, 8). In mice, surface expression of CD49b and expression of the transcription factor Eomesodermin (EOMES) merge cells under the umbrella of conventional NK cells. In contrast, CD49a expression and the absence of EOMES expression assigns cells to the ILC1 lineage (7, 9). However, to add a layer of complexity it was shown that CD49a expression can be induced on conventional mouse NK cells *in vivo* upon viral (10) and parasite infection (11) and in the tumor microenvironment (12, 13). Treatment of mouse splenic NK cells with IL-2 and TGF-β induces the expression of ILC1-associated markers, such as CD49a and TRAIL (12). On the other hand, expression of EOMES under the control of the *Tbx21* (T-BET) locus induces ILC1s to acquire an NK cell-like phenotype (14).

The high plasticity within group 1 ILCs and the reversible trans-differentiation of group 2 and 3 ILCs into ILC1s (15) complicate the task to dissect the impact of aberrant cytokine signaling or expression of signaling molecules on those cells. It might thus be necessary to re-evaluate some previously published literature on NK cells to determine whether conventional NK cells and/or ILC1s have been analyzed.

## NK Cell Development and Maturation

NK cells originate from common lymphoid progenitors (CLPs) in the bone marrow and may traffic to secondary lymphoid tissues, where they undergo terminal maturation and exit to the circulation (16, 17). The α-lymphoid progenitor (α-LP) and the early ILC progenitor (EILP) are the first progenitors with restricted lineage potential for all ILC subsets (18, 19). Downstream of EILPs are NK precursors (NKPs) giving rise to conventional NK cells and common helper-like innate lymphoid precursors (CHILPs), the ancestors of all other ILC subsets including ILC1s (15). The most distinct characteristic of NKPs is the acquisition of CD122 (IL2Rβ) expression, which is pivotal in the transduction of IL-15 signals via JAK1/3 and STAT5. Loss of one of these components unequivocally precludes NK cell development (20–23). This already highlights the central role of the JAK/STAT signaling cascade in NK cell development and maturation.

Human NK cells, classified as CD3^−^CD56^+^NKp46^+^ cells, can be further subdivided based on the expression of the low affinity Fc-receptor CD16 in CD56^bright^CD16^−^ and CD56^dim^CD16^+^ cells. CD56^bright^CD16^−^ NK cells are more responsive to stimulation by inflammatory cytokines and are thought to be immature precursors of CD56^dim^CD16^+^ mature NK cells, which show a higher cytotoxic capacity. The development of human NK cells can be stratified to five stages (16). The final maturation of human NK cells is accompanied by the loss of CD94/NKG2A and CD226 (DNAM1) expression, the acquisition of killer immunoglobulin-like receptors (KIRs) and CD57, and the change in the expression pattern of homing molecules such as CD62L (24, 25). Recently though, several studies have challenged this traditional model and suggested that CD56^dim^CD16^+^ and CD56^bright^CD16^−^ NK cells may arise from separate lineages (26).

Mouse NK cells are defined as CD3^−^CD49b^+^NKp46^+^ cells and in C57BL/6 mice additionally NK1.1^+^. Their maturation in the periphery is associated with the upregulation of CD11b, CD43, KLRG1, and Ly49 receptors, and the downregulation of CD27 (17). Although the acquisition or loss of these surface markers is happening on a continuous scale, it has become customary to distinguish three subsets of immature (CD27^+^CD11b^−^), semi-mature (CD27^+^CD11b^+^) and mature (CD27^−^CD11b^+^) NK cells (27, 28).

In general, compared to their more immature counterparts, mature NK cells produce less cytokines, show a reduced proliferative capacity, but become more cytotoxic against target cells. However, in the process of terminal differentiation NK cells gradually lose their effector functions as well as the expression of the activating receptor DNAM1 (24, 28).

## JAK/STAT Signaling

**Figure 1:**
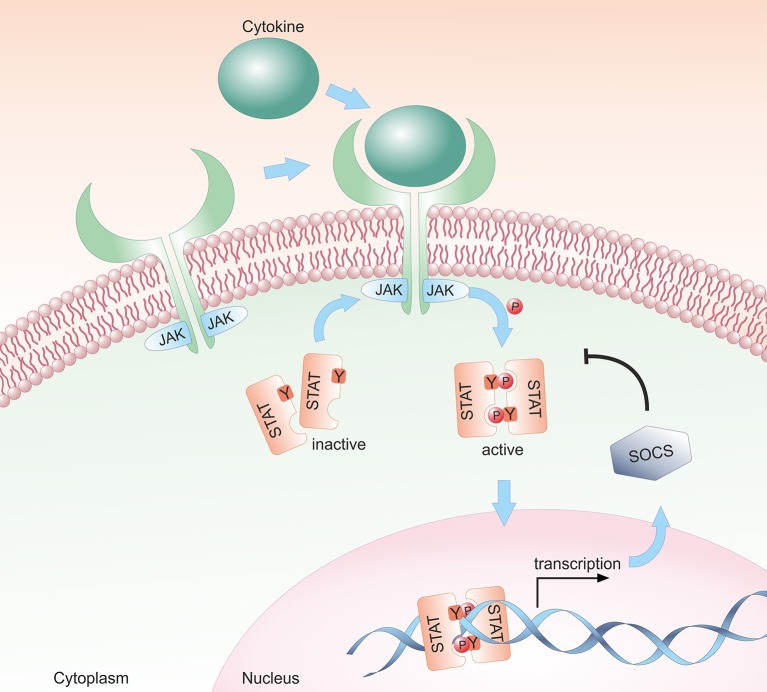
Schematic representation of the canonical JAK/STAT signaling pathway. The JAK/STAT pathway transmits extracellular cytokine signals to the nucleus. Upon binding of a cytokine to its transmembrane receptor, receptor-associated JAKs are activated and phosphorylate STAT proteins. Activated STAT proteins translocate as either homo- or hetero-dimers to the nucleus and modulate target gene transcription. In a negative feedback loop, SOCS proteins are expressed and inhibit the JAK/STAT signaling cascade by suppressing JAK kinase activity, by competing with STAT proteins for binding to the receptor and/or by proteasomal degradation of the proteins.

**Figure 2:**
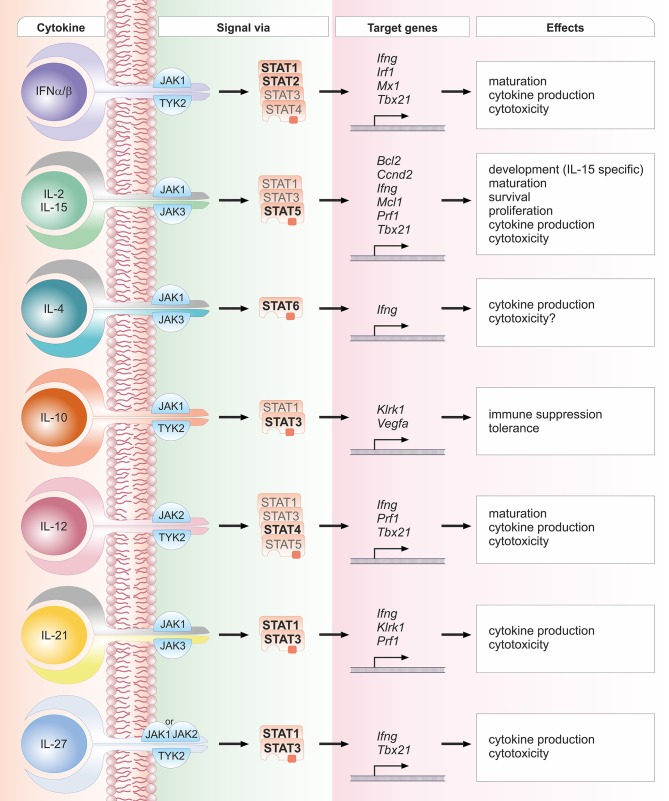
Schematic overview of crucial cytokines in NK cell biology, their associated JAK and STAT proteins, exemplary target genes and biological effects. One cytokine can lead to the activation of several STAT proteins. STAT proteins predominantly activated by the respective cytokine are depicted in bold font; STAT proteins that have been reported to be activated to a lesser extent are depicted in light font. The details and references for distinct cytokine signaling cascades and the functional responses can be found in the corresponding sections of the main text.

Most cytokines that influence group 1 ILC development or functions signal via the Janus kinase / signal transducer and activator of transcription (JAK/STAT) pathway (see Figure 1). Depending on the cell type, developmental status and microenvironment, JAK/STAT signaling contributes to the regulation of differentiation, proliferation, migration, survival or cytotoxicity in response to more than 50 cytokines, growth factors and hormones (29–31). Many of these cytokines are crucial for NK cells; their signal transduction and downstream effects are summarized in Figure 2. To allow this enormous complexity, the JAK/STAT signaling cascade transports extracellular signals from the cell membrane to the nucleus via various steps. In the canonical signaling cascade, extracellular binding of a cytokine to its corresponding multimeric receptor leads to conformational changes of the receptor chains. Receptor-associated JAK kinases come into close proximity, and sequentially phosphorylate each other and the intracellular portion of the receptor. This creates docking sites for STAT proteins that are recruited to the receptors and phosphorylated on their tyrosine residues by JAK kinases. STAT phosphorylation provokes detachment from the receptor, the formation of homo- or hetero-dimers with other STAT proteins and nuclear translocation. In the nucleus STATs regulate target gene transcription by binding to promotor or enhancer motifs or other non-coding intra- and intergenic regions (29–31) (see Figure 1). In addition, several non-canonical pathways have been described; these include kinase-independent functions of JAKs, the formation of higher order STAT tetramers or multifactorial complexes with other transcription factors, and pathways building on unphosphorylated STAT proteins (U-STATs) (31, 32). In NK cells, non-canonical functions have so far been described for TYK2, STAT1 and STAT5 (see below).

**Figure 3:**
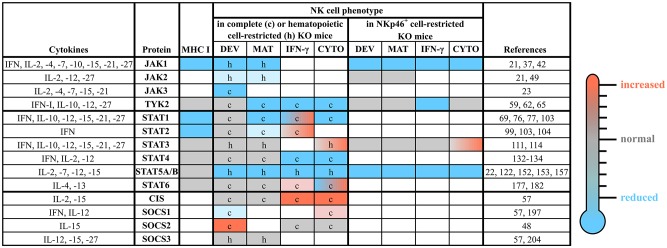
The roles of distinct JAK/STAT and SOCS family members in NK cells. NK cell development, maturation and function are tightly regulated by a plethora of cytokines, which most prominently use the JAK/STAT pathway for their signal transduction. This figure summarizes the available literature about each member of the JAK/STAT signaling pathway and some of their negative regulators (SOCS1-3 and CIS), relevant upstream cytokines and the NK cell phenotypes observed in complete or conditional knockout mice. The individual cells are coded by color: compared to wild-type reduced (blue), unchanged (gray) or increased (red); blank cells indicate not determined yet. c, complete knockout mice; CIS, cytokine induced SH2-containing protein; CYTO, cytotoxicity; DEV, development; h, hematopoietic cell-restricted knockout mice; IFN, interferon; IL, interleukin; IFN-γ, IFN-γ production; JAK, Janus kinase; KO, knockout; MAT, maturation; MHC, major histocompatibility complex; SOCS, suppressor of cytokine signaling; STAT, signal transducer and activator of transcription; TYK2, tyrosine kinase 2.

The JAK/STAT pathway is highly conserved among species. Mammals express four members of the JAK family (JAK1-3 and TYK2) and seven STAT proteins (STAT1-4, STAT5A, STAT5B, and STAT6). STAT5A and STAT5B are highly homologous but encoded by distinct genes located on the same chromosome directly adjacent to the *Stat3* gene locus indicating that these three genes derived from the duplication of a common primordial gene (33, 34). Although distinct members of the JAK/STAT cascade share high homology, their specific functions vary considerably. Gene-targeted mice have deepened our understanding of distinct roles of individual JAK and STAT proteins (see Figure 3). Deficiency of *Jak2* (35) and *Stat3* (36) precludes embryonic development, whereas *Jak1-* (37) and *Stat5a/b*-deficiencies (38) lead to perinatal lethality. Loss of the other members of the JAK/STAT pathway does not interfere with viability of the animals, but reveals distinct phenotypes including the absence of lymph nodes and/or high sensitivity to infections (39).

### JAK1

JAK1 is involved in the signal transduction of several cytokines crucial for NK cell biology, for instance the IL-2 family cytokines including IL-2, IL-4, IL-7, IL-9, IL-15, and IL-21. Most importantly, IL-15 represents the major cytokine regulating NK cell development, maturation and function (17, 40). Additionally, as the major component downstream of IFNs and IL-10, JAK1 plays a pivotal role in NK cell biology (41). It should also be mentioned that JAK1 associates with the IL-4 receptor family (transmitting IL-4 and IL-13 signals) and the gp130 receptor family (transmitting, e.g., IL-6, IL-11, IL-27, LIF, and OSM signals) (41).

Given the fundamental role of IL-15 and other IL-2 family cytokines transmitted via the common γ (γc) receptor, it is not surprising that complete loss of *Jak1* leads to perinatal lethality in mice, accompanied by a strong reduction in the number of thymocytes and B cells (37). These observations were recently confirmed in adult mice: inducible deletion of *Jak1* leads to impaired hematopoietic stem cell homeostasis and a pronounced decrease in immature B220^+^ NK cells (42).

Using mice with NKp46^+^ cell-specific deletion of *Jak1* uncovered the crucial role of JAK1 in NK cell development and survival (21). *Jak1* deficiency reduces the numbers of NK cells and ILC1s in a dose-dependent manner. This indicates that other JAK family members fail to compensate for the loss of JAK1. The consequences of *Jak1* deletion within the NK cell compartment exceed the effects seen upon loss of the JAK1 downstream effector STAT5 (21, 22). Different half-lives of JAK1 and STAT5 proteins may contribute to the difference in NK cell frequency. One may also reason that the more pronounced depletion of NK cells results from the combined loss of STAT3 and STAT5-mediated signals in *Jak1*-deficient animals.

To the best of our knowledge, no reports on *JAK1*-deficient individuals exist so far, suggesting that like in mice, it might lead to embryonic lethality in humans. A patient harboring a biallelic *JAK1* germline mutation leading to a partial loss of kinase activity has been reported. The resulting functional impairment was associated with a mild immunodeficiency, recurrent atypical mycobacterial infections and early onset metastatic bladder carcinoma (43).

### JAK2

JAK2 is a critical mediator of growth hormone (GH), erythropoietin and IFN-II signaling and thus plays a pivotal role in hematopoiesis (35, 44). In both NK and T cells, IL-2 signals via JAK1/3 and STAT1/3/5 inducing NK and T cell proliferation and enhancing NK cell cytotoxicity. However, unlike in T cells, in NK cells IL-2 additionally activates JAK2 and STAT4 (45). JAK2 in combination with TYK2 mediates the signal transduction of the IL-12 family members: IL-12 activates STAT4 and to a lesser degree STAT1, STAT3 and STAT5 (46); IL-23 activates mainly STAT3 and STAT4, and IL-27 signals mainly via STAT1 and STAT3 (47). Although it was assumed that IL-15 signals exclusively via JAK1/3, a recent study described an IL-15-mediated JAK2 activation in murine NK cells (48).

Germline deletion of *Jak2* results in embryonic lethality at day 12.5 due to impaired hematopoiesis (35). Studies using *Jak2*-conditional knockout mice uncovered a mild defect in NK cell maturation in the absence of JAK2 in the hematopoietic system (49). In line, treatment of mice with the JAK2 inhibitor BSK805 reduces NK cell numbers due to decreased proliferation and an immature maturation profile resulting in an increased metastatic burden (49). In contrast, deletion of *Jak2* in mature NK cells does not impact on NK cell numbers or maturation (21). It is conceivable that JAK2 is crucial for development and maturation of early NK cell progenitor stages in the bone marrow. Alternatively, JAK2-inhibition or deletion may interfere with other cell types to alter NK cells extrinsically by changing the cytokine milieu. JAK2 has been reported to be required for the development of dendritic cells (DCs) (50), which are potent producers of IL-15 and thus indispensable for proper NK cell priming (51). DC-mediated NK cell priming is potentially impaired upon JAK2 inhibition or deletion. Support for this concept stems from the observation that IL-15 treatment overcomes the JAK2 inhibitor-mediated increase of tumor metastasis (49).

### JAK3

Together with JAK1, JAK3 transmits signals downstream of the γc cytokines IL-2, IL-4, IL-7, IL-9, IL-15 and IL-21 resulting in phosphorylation of STAT1, STAT3, STAT4, STAT5 and STAT6 (41, 52). *Jak3*^−/−^ mice are immunocompromised, display severe developmental defects in the lymphoid lineage and lack NK, T and B cells (23, 53). The *in vivo* administration of Tofacitinib, which predominantly inhibits JAK3 and to a lesser degree JAK1 and JAK2, depletes all NK cell subsets in the periphery of rhesus macaques (54). Likewise, human patients harboring *JAK3* mutations suffer from severe combined immunodeficiency (SCID) lacking NK and T cells (55). A previous study in mice having a spontaneous *Jak3* mutation disclosed the association of an impaired JAK3 signaling with a differentiation block of NK and ILC1s at the pre-NKP and ILCP stage (56). In summary, these findings define a non-redundant role of JAK1 and JAK3 for NK cell development and the differentiation of EILPs.

Interestingly, quantitative mass spectrometry analysis in mature NK cells demonstrated a predominance of JAK1 protein compared to JAK3 (57). It was further proposed that JAK1 dominates JAK3 in the signal transduction of γc cytokines. While loss of the JAK1 kinase function completely abrogates downstream signals, loss of the kinase activity of JAK3 in human cell lines only diminishes STAT5 phosphorylation. It was thus suggested that JAK3 functions by activating JAK1, but does not directly induce STAT5 phosphorylation (58). The details of the molecular interactions in NK cells remain to be determined. It is currently unclear what effect the conditional deletion of *Jak3* in mature NK cells will have and how it will affect their proliferation and effector functions.

### TYK2

TYK2 associates with the IFN-I (IFNAR1), IL-10Rβ, IL-12Rβ1, and IL13Rα1 receptors and is thus involved in the signal transduction of a large number of cytokines including IFN-I, IL-10, IL-12, IL-23, and IL-27 (46). Despite its broad activity, *Tyk2*-deficiency does not preclude survival of mice (59, 60).

NK cells derived from *Tyk2*^−/−^ mice display impaired IL-12-mediated signaling resulting in reduced STAT1, STAT3, and STAT4 activation (61, 62). Although the development of NK cells in the bone marrow is unaltered, the final maturation in the periphery is severely impaired as evidenced by fewer CD27^−^CD11b^+^ and KLRG1^+^ cells in *Tyk2*-deficient mice. This translates into impaired IFN-γ production and cytolytic responses (62) in line with the involvement of STAT4 in the regulation of IFN-γ and perforin expression, respectively (63, 64). Mice expressing a kinase-inactive version of TYK2 (*Tyk2*^*K*923*E*^) show a milder defect in NK cell maturation and cytotoxicity compared to *Tyk2*^−/−^ mice, indicating that TYK2 has kinase-independent functions (62). Using mice with NKp46^+^ cell-specific deletion of *Tyk2* revealed that the impact of TYK2 on NK cell maturation and tumor surveillance is cell-extrinsic and depends on the presence of TYK2 in dendritic cells (65). Accordingly, the defects in NK cell maturation and cytotoxicity related to *Tyk2-*deficiency are reversed upon treatment with recombinant IL-15/IL-15Rα. However, NK cell-intrinsic TYK2 is required for IL-12-induced IFN-γ production and the defense against *Listeria monocytogenes* (65).

*Tyk2*-deficient mice and patients with autosomal recessive *TYK2* mutations are susceptible to infections. NK cells from *TYK2*-deficient patients have an impaired, albeit not completely abrogated, IL-12-mediated IFN-γ production (60). This may explain why their susceptibility to viral infections is less severe when compared to patients harboring a *STAT1-*deficiency (66). A thorough analysis of peripheral NK cell maturation in the reported *TYK2*-deficient patients is pending.

### STAT1

STAT1 is the predominant transcription factor activated by IFNs, irrespective of the subtype. Whereas, type II IFN (IFN-γ) induces the homo-dimerization of STAT1, type I IFN (IFN-α/β/ε/κ/ω) and type III IFN (IFN-λ 1-3) signaling triggers the formation of the ISGF3 complex consisting of STAT1, STAT2, and IRF9. In addition, STAT1 transmits signals from IL-6, IL-10, IL-12, IL-15, IL-21, IL-27, and IL-35 (31, 67–71).

IFNs play a pivotal role in NK cell maturation, as they provide the necessary signals for IL-15 trans-presentation by DCs (51) and MHC class I expression (37, 72, 73). *Stat1*-deficient NK cells show profound defects in NK cell maturation, cytokine-induced IFN-γ production, cytolytic capacity and memory formation (69, 74–77). In line, NK cells from *Ifnar1*-deficient animals display an immature phenotype (78) as well as defects in basal (79) and virus-induced cytotoxicity (80). The maturation defect seen in complete *Ifnar1*-knockout mice was not recapitulated upon NKp46^+^ cell-specific *Ifnar1* gene deletion (78), suggesting that NK cell-extrinsic factors, such as the presentation of MHC class I and/or IFN-mediated trans-presentation of IL-15, play a crucial role in proper NK cell licensing and maturation. Accordingly, the transfer of *Stat1*-deficient bone marrow into wild-type mice provided sufficient signals for proper NK cell maturation *in vivo* (76).

In addition to IFNs, the STAT1/3 activating (70) cytokine IL-21 is known to drive the maturation of mouse and human NK cells (81, 82). Recombinant IL-21 treatment not only increases CD8^+^ T cell functions (83) but also the cytotoxicity and cytokine production of NK cells by inducing the expression of perforin and IFN-γ, respectively (82, 84). However, mice lacking the IL-21R do not display any defect in NK cell numbers or maturation (85) arguing against a profound effect of IL-21 on NK cell maturation under homeostatic conditions *in vivo*.

Analogous to IL-21, IL-27 signals mainly via STAT1 and STAT3 (47, 86). Whereas, IL-27 treatment alone does not have a major impact on NK cells, co-stimulation with IL-27 and IL-2, IL-12 or IL-18 leads to enhanced NK cell activation, cytokine production and cytotoxicity (47, 86–88). Accordingly, loss of IL-27R in mice leads to reduced NK cell-mediated IFN-γ production and T-BET expression after influenza virus infection (89). *Il27ra*-deficient mice are characterized by an unusual maturation profile, represented by fewer mature NK cells in the bone marrow, while more mature NK cells are found in the spleen (89). This phenotype does not reflect the situation in *Stat1*-deficient mice and may suggest an altered dissemination of NK cells rather than a maturation defect.

However, *Stat1-Y701F* knock-in mice lacking the tyrosine residue essential for STAT1 translocation and transcriptional activity do mirror the impaired maturation phenotype of NK cells seen in *Stat1*^−/−^ mice (90). Although the function of STAT1 was considered to depend on the tyrosine phosphorylation, *Stat1-Y701F* expressing NK cells are more cytotoxic against tumor cells than *Stat1*^−/−^ NK cells. A novel non-canonical function of STAT1 at the immunological synapse of NK cells regulating tumor surveillance and cytotoxicity may account for that effect (90). Whereas STAT1-Y701 phosphorylation is triggered by cytokine stimulation, non-stimulated primary mouse NK cells display a constitutive CDK8-mediated phosphorylation of STAT1-S727 (69). The introduction of a point-mutation (*Stat1-S727A*) that prevents the serine phosphorylation event results in NK cells that produce less IFN-γ upon stimulation, and have a mild defect in KLRG1 and NKG2A/C/E expression. Nevertheless, these *Stat1-S727A* NK cells show enhanced cytotoxicity against tumors *in vitro* and *in vivo*, which correlates with increased perforin and granzyme B levels (69), once more highlighting the existence of non-canonical STAT signaling (32).

Like *Tyk2*-deficient mice, *Stat1*-deficient mice are highly susceptible to bacterial and viral infections (80, 91–93). Biallelic *loss-of-expression* or *loss-of-function* (*LOF*) *STAT1*-deficiency in humans is detrimental, with most patients succumbing to lethal infections with mycobacteria or herpes simplex virus 1 (HSV-1) encephalitis before the age of two years (66), which is accompanied by a profound effect on NK cell cytotoxicity (94). Unexpectedly, *STAT1 gain-of-function (GOF)* mutations are likewise associated with increased susceptibility to infectious diseases, such as chronic mucocutaneous candidiasis, bacterial and viral infections, autoimmune diseases and even cancer (95). *STAT1 GOF* patients have fewer and highly immature CD56^dim^ NK cells in the periphery showing reduced cytotoxicity, IFN-γ production and cytokine-induced proliferation (96, 97). This defect was partially rescued by treatment with the JAK1/2 inhibitor ruxolitinib (96) and improved the patients' clinical picture (98). The mechanism of how *STAT1 GOF* mutations result in hyporesponsive NK cells is not fully understood, but it was paralleled by decreased activation of STAT5 (96), which is a master regulator for NK cell functions. These observations indicate that STAT1 signaling needs to be tightly controlled and neither reduced nor excessive pathway activation is beneficial for NK cell maturation and function.

### STAT2

STAT2 together with STAT1 and IRF9 are activated in response to IFN-I and IFN-III. This turns STAT2 into a crucial mediator of antiviral defense. Depending on the viral challenge, *Stat2*^−/−^ mice are more (99, 100) or less (101, 102) susceptible to infection compared to *Stat1*^−/−^ mice. In the course of lymphocytic choriomeningitis virus (LCMV) infections, STAT1 and STAT2 are both required for optimal viral control, but STAT2 plays a subordinate role compared to STAT1: although both *Stat1-* and *Stat2*-deficient NK cells produce increased amounts of IFN-γ early after LCMV infection, this exclusively drives bodyweight loss in the absence of STAT1, but not STAT2 (103). *Stat2*^−/−^ mice are highly susceptible to MCMV infection and succumb within the first week after infection (100). In line with a crucial role of IFN-I signaling in NK cell expansion and memory formation in the context of MCMV, NK cells from *Stat1-, Stat2-*, and *Irf9*-deficient mice are defective in their ability to expand (74, 104). As shown in *Stat1*^−/−^ and *Ifnar1*^−/−^ mice, also *Stat2*^−/−^ NK cells have a defect in NK cell maturation, which could be rescued in bone marrow chimeras (104), again suggesting an NK cell-extrinsic role of IFN-I in NK cell maturation.

*STAT2*-deficient human patients present a higher incidence of distinct viral infections with astounding variation ranging from asymptomatic adult carriers of the mutation to infants succumbing to viral illness. In particular, fatal prolonged febrile encephalitic illness following measles/mumps/rubella vaccination has been reported in six vaccinated children with a *STAT2* deficiency (105–107). Unlike STAT1, STAT2-mediated signaling seems to be dispensable for host defenses against most viral childhood diseases such as respiratory syncytial virus bronchiolitis or HSV-1 as well as infections with intracellular bacteria. This can be partially explained by an unaltered response to IFN-II in *STAT2*-deficient patients, and the observation that depending on the bacterial infection IFN-I can play adverse roles (108, 109).

Besides its role in antiviral responses, IFN-I has been implicated in anti-tumor immunity. While the contribution of STAT2 for T cell-mediated tumor surveillance has been unequivocally documented (110), the role of STAT2 in NK cell-mediated tumor surveillance is still enigmatic.

### STAT3

Cytokines such as IL-2, IL-10, IL-12, IL-15, IL-21, IL-27, and IFN-I induce STAT3-Y705 phosphorylation in NK cells (111, 112). While most of these cytokines positively regulate NK cell maturation and/or activation, IL-10 is classified as immunosuppressive cytokine (113).

Several studies reported constitutive STAT3 phosphorylation of tumor-infiltrating immune cells including NK cells (114, 115). STAT3 phosphorylation is considered to be driven by inflammatory and immunosuppressive cytokines and growth factors produced by both tumor and tumor-infiltrating cells including IL-6, IL-10, or VEGF-A. STAT3 activation in the tumor stroma has been associated with an impaired tumor immune surveillance of both NK and CD8^+^ T cells (116, 117). High IL-10 levels in the liver also dampen hepatic NK cell responses and restrain the expression of Ly49 receptors (118). In light of recent advances in the discrimination of NK cells and ILC1s, these observations could potentially indicate a specific role of IL-10 in ILC1s, which lack most of the Ly49 receptors (119). This suppressive role is of particular importance in the liver, where IL-10 ensures that liver NK cells/ILC1s remain immune-tolerant, but is undesirable in the context of tumor surveillance (113, 115). Under certain conditions NK cells themselves (120) and the recently described regulatory ILCs have been reported to produce IL-10, which inhibits cytokine-induced IFN-γ production of ILC1s (121).

Studies in mice with constitutive or NKp46^+^ cell-specific *Stat3*-deficiency indeed show that STAT3 suppresses NK cell-mediated tumor surveillance in melanoma and leukemia models (111, 114). Loss of *Stat3* does not alter classical NK cell maturation but is paralleled by increased expression of the activating receptor and maturation marker DNAM1 as well as increased expression of STAT5 and its downstream targets perforin and granzyme B (111). It is thus attractive to speculate that STAT3 represses STAT5-mediated signaling in wildtype NK cells. As described below, STAT5 represents a master regulator of NK cell function. The fact that IL-15 stimulation induces both STAT3 and STAT5 activation in NK cells (111, 113) endorses the hypothesis that STAT3 is crucial to control IL-15/STAT5-mediated NK-cell cytotoxicity to prevent detrimental hyperactivity. This concept warrants testing of a combined treatment with IL-15 and anti-STAT3 inhibitors in the context of anti-cancer immunotherapy.

Alternatively, STAT3 acts downstream of the cytokine IL-10 (111), which has been shown to transcriptionally induce the tumor promoting factor VEGF-A in NK cells (122). It is thus attractive to speculate that STAT3 activation in NK cells promotes tumor progression by dampening their cytolytic activity and driving tumor angiogenesis.

Besides suppressing cytotoxicity, STAT3 regulates the expression of the activating receptor NKG2D. IL-10 and IL-21 treatment induces NKG2D expression in a STAT3-dependent manner in human and mouse NK cells (71, 123). In line, human NK cells derived from hyper-IgE syndrome patients carrying *STAT3 LOF* mutations show a pronounced decrease of NKG2D expression (71).

*STAT3 GOF* mutations in NK cells can be found in patients with chronic lymphoproliferative disorders of NK cells (CLPD-NKs) (124) as well as aggressive NK cell leukemia (125) and extranodal NK/T-cell lymphoma (NKTCL) (126, 127). The identified *STAT3* mutations enhance the levels of phosphorylated STAT3 protein and provide a growth advantage to the affected cells. These findings support the concept that STAT3 has an oncogenic potential in NK cells and highlight the importance of tight controls and negative feedback regulators.

### STAT4

In contrast to other immune cells such as CD8^+^ T cells, IL-2 stimulation induces JAK2 and STAT4 activation in NK cells and enhances IL-12 signaling by upregulating the expression of the IL-12R (128, 129). IL-12 is the main driver of STAT4 activation and crucial for IFN-γ production in NK cells and ILC1s (129–131). Under steady-state conditions, *Stat4*- as well as *Il12r*-deficient mice harbor an unaltered NK cell repertoire in the periphery. Due to its rather restricted action downstream of IL-12, *Stat4*-deficiency in mice manifests in reduced IL-12-induced NK cell proliferation, IFN-γ production and cytotoxicity (132, 133). This can be explained by the fact that STAT4 regulates the induction of T-BET, a transcription factor important for NK and ILC1s that induces the transcription of important key players of the cytotoxic machinery, such as IFN-γ, granzyme B and perforin (131, 134). STAT4 and T-BET are also necessary for the generation and maintenance of MCMV-specific memory NK cells (135, 136). In line with the lessons learnt from mice, a heterozygous missense mutation in *STAT4* leading to a defect in IL-12-dependent IFN-γ immunity was identified in two patients suffering from acute chronic fungal infections (137).

IL-12 also has a unique and detrimental role in adipose tissue, as diet-induced obesity is associated with IL-12 production and the proliferation and subsequent accumulation of adipose-resident ILC1 and NK cells. IL-12/STAT4 signaling is required for the increased proliferation and IFN-γ production of all group 1 ILC subsets in the adipose tissue driving M1 macrophage polarization and obesity-associated insulin resistance (138).

Apart from IL-12, IFN-I has been reported to induce phosphorylation and dimerization of STAT4 amongst all other STAT proteins (139). NK cells have particularly high basal STAT4 levels pre-bound to IFNAR1 (103). During the early phase of viral infections, STAT4 becomes activated initiating a fast IFN-γ response followed by STAT1 activation, which replaces STAT4 at the IFNAR receptor decreasing the ability to produce IFN-γ (103). These data exemplify how one cytokine activates several STAT molecules enabling a tight regulation of cellular responses.

### STAT5A and STAT5B

Of all STAT proteins, STAT5 is the major regulator of NK cell development, maturation, survival and function and is activated by cytokines such as IL-2, IL-7 and IL-15. Compared to IL-15, IL-2, and IL-7 play a minor role in the development and survival of NK cells and ILC1s (140, 141). STAT5 is also implicated in the development, survival and memory formation of CD8^+^ T cells, which is regulated by IL-2, IL-7, and IL-15 signaling (142, 143). IL-2 plays a crucial role in activating CD8^+^ T and NK cells against target cells *in vivo* (144, 145) and it is therefore commonly added to *in vitro* culture systems. Although mouse NKPs express high levels of CD127 (IL-7Rα) (146), NK cell development and function are unaltered in the absence of IL-7 signaling in mice (147). The only exception are thymic NK cells, whose development depends on IL-7 and the transcription factor GATA3 (148). By contrast, in humans IL-7 controls the survival of immature CD56^bright^ NK cells (149).

Knock-out mice lacking IL-15 or its receptor subunits are devoid of NK cells proving the indispensable role of IL-15 for NK cell development (141, 150, 151). IL-15 trans-presentation by DCs is crucial to prime NK cell maturation and function (51). As STAT5 is a critical transcription factor downstream of IL-15, impaired STAT5 signaling impacts strongly on NK cell viability and function (152, 153).

In general, STAT5 is an umbrella term for two distinct transcription factors: STAT5A and STAT5B sharing 96% sequence homology. Despite largely redundant functions, several non-redundant and tissue-specific roles have been described (154–156). *Stat5a/b*-deficiency in mice is perinatally lethal due to anemia and hematopoietic failure (38). Early on, STAT5 has been described to be essential for NK cell development, as the first STAT5 knockout mice that express an N-terminally truncated version of *Stat5a/b* are viable but devoid of peripheral NK cells (153). Single knockout mice for *Stat5a* or *Stat5b* verified the impact of STAT5 for NK cell development, maturation and cytoloytic capacity also indicating non-redundant functions of STAT5A and STAT5B. STAT5B is the dominant isoform for NK cells as its deletion has a significantly larger impact than deletion of STAT5A (152, 157). This is explained by a higher abundance of STAT5B over STAT5A transcripts in NK cells (157). Mice expressing only one allele of either *Stat5a* or *Stat5b* (*Stat5a*^+/−^*Stat5b*^−/−^ and *Stat5a*^−/−^*Stat5b*^+/−^*)* have drastically diminished numbers of NK and ILC1 progenitors, splenic NK cells as well as intestinal and liver NK cells (157). Liver-resident ILC1s and bone marrow NK cells are less sensitive to reduced STAT5 expression levels. STAT5 was also verified as an upstream regulator of the transcription factor T-BET (122, 158) and a recent study showed that both transcription factors co-localize throughout the genome (157).

The cell-intrinsic role of STAT5 in NK cells was studied using mice where *Stat5a/b* deletion is restricted to NKp46^+^ cells. This results in a severe reduction of peripheral NK cells (22) as NK cell survival relies on the expression of anti-apoptotic STAT5 target genes such as *Mcl1* or *Bcl2* (122, 159). The residual NK cells found in the bone marrow of *Stat5*^*fl*/*fl*^*Ncr1iCre*^*Tg*^ mice harbor an immature phenotype and a major developmental block at the NKP stage (22). Enforced expression of the anti-apoptotic factor Bcl-2 rescues survival of *Stat5a/b*-deficient NK cells, but does not allow proliferation, maturation and reconstitution of effector functions (122). Apart from the central role of STAT5 driving the expression of transcription factors pivotal for NK cell development (ID2, EOMES and T-BET) and regulating the expression of crucial effector molecules (perforin, granzymes and IFN-γ), STAT5 has been reported to suppress the expression of the pro-angiogenic factor VEGF-A in NK cells (122). Further research is necessary to verify if STAT5 directly acts as a transcriptional suppressor or competes with the binding of other activating transcription factors.

Decidual NK cell-derived VEGF-A has a positive impact on neo-angiogenesis and placenta development during pregnancy (160–162). In contrast, the expression of VEGF-A in tumor-infiltrating NK cells promotes tumor formation (122). VEGF-A-secreting tumor-associated NK cells have also been reported in patients and are associated with poor disease outcome (163–165).

In line with observations in mice, patients with a *STAT5B LOF* mutation harbor significantly reduced NK cell numbers (166–168). *STAT5 GOF* mutations are found in malignancies of innate and innate-like lymphoid cells (125, 127, 169) and drive tumorigenesis in mouse NKT cells (170).

STATs in general, but STAT5 proteins in particular, are known to form higher order tetramers. A recent study highlighted the importance of STAT5 dimers for NK cell development, while the formation of STAT5 tetramers is a prerequisite for proper NK cell maturation and survival. The authors speculate that interfering with STAT5 tetramer formation could be used therapeutically to restrict the growth of NK cell leukemia and lymphomas (171).

To summarize, STAT5 is a master regulator of NK cells ensuring their development and survival and regulating maturation, proliferation, cytotoxicity and their precarious production of VEGF-A.

### STAT6

STAT6 is activated by IL-4 and IL-13 and is involved in Th2 polarization and the development of allergic inflammation (172). Allergies and IL-4 signaling have been suggested to protect from cancer development. Indeed, IL-4 overexpression in combination with phthalic anhydride-induced allergy induction in mice enhances NK cell activity and reduces tumor burden (173). The effect of IL-4 on NK cells is highly controversial, as it was shown that IL-4 treatment of purified NK cells diminishes their cytotoxic capacity (174), while it enhances NK cell cytotoxicity and IFN-γ production when applied *in vivo* (175). IL-4 synergizes with IL-12 and/or IL-2 to induce IFN-γ production, which was shown to be partially dependent on STAT6 (176). *In vitro* stimulation of mouse NK cells with a mixture of phorbol 12-myristate 13-acetate (PMA), ionomycin, IL-2, IL-4, and anti-IFN-γ mAb induces IL-5 and IL-13 production in a STAT6-dependent manner (177). Also human NK cells possess the ability to produce IL-5 and IL-13 upon IL-4 stimulation (178, 179). In various allergic diseases, such as asthma and allergic rhinitis, NK cell-derived Th2 cytokine production contributes to eosinophil infiltration and thereby promotes allergic inflammation (180, 181). Although the involvement of STAT6 in this signaling is highly probable, it still awaits formal proof.

It was previously shown that loss of STAT6 in mice does not impact on NK cell development or maturation (177). However, *Stat6*-deficiency is associated with higher cytotoxic activity of NK cells and increased resistance to ectromelia virus infection (182). The seeming opposing results showing enhanced cytotoxicity of IL-4/STAT6-activated as well as STAT6-deficient NK cells certainly call for a more detailed analysis of the role of STAT6 in NK cells in the context of anti-tumor and anti-viral immunity.

## SOCS Proteins

The family of suppressor of cytokine signaling (SOCS) proteins has eight members including SOCS1-7 and CIS which represent important negative regulators of the JAK/STAT signaling pathway (183, 184). SOCS proteins are characterized by a central SH2 domain and an extended SH2 sub-domain, a highly conserved C-terminal SOCS box and a variable N-terminal region. SOCS proteins exert their eponymous function by three means: (i) Via their SH2 domain, SOCS proteins bind to phosphotyrosine residues on cytokine receptors thereby competing with STAT binding and activation. (ii) Via the SOCS box they recruit an E3 ubiquitin ligase complex that leads to proteasomal degradation of signaling molecules including cytokine receptors and JAK kinases. (iii) The N-terminal domain of SOCS1 and SOCS3 contains a kinase inhibitory region serving as pseudo-substrate for JAKs consequently blocking their activity (183, 184).

Immunomodulatory effects of SOCS proteins on NK cells have been reported and suggest them as attractive candidates for immunotherapies (185). SOCS1, 2, 3 and CIS are rapidly induced upon cytokine (57, 186) or GH (187) stimulation. In contrast, little is known about the residual family members SOCS 4-7 that are constitutively expressed in unstimulated cells (185). SOCS4 and SOCS5 are crucial regulators of anti-viral immunity in the context of influenza infection (188, 189). SOCS6 negatively regulates JAK/STAT3 signaling and is epigenetically silenced in NK cell lymphomas (190). *Socs7*^−/−^ mice suffer from a severe cutaneous disease due to hyperactive mast cells and the increased production of pro-inflammatory cytokines (191).

### CIS

The cytokine induced SH2-containing protein (CIS, *Cish*) is induced by IL-2 and IL-15 and provides a negative feedback loop to inhibit JAK/STAT5-mediated signaling in NK cells (57). CIS interacts with JAK1 to target it for proteasomal degradation and thereby abrogates IL-15-induced signaling. In line with the crucial function of the IL-15/STAT5 axis for NK cell biology, hyperactive IL-15 signaling in *Cish*^−/−^ mice translates to enhanced NK cell proliferation and cytotoxic function. This ultimately leads to resistance toward experimental metastasis (57) and chemically-induced sarcoma (192). *Cish*^−/−^ mice react to IL-2 treatment with a further decrease of tumor burden in models that are usually unaffected by IL-2 treatment. Additive effects were also observed when CIS-deficiency was combined with targeted immunotherapies such as BRAF and MEK inhibitors, immune checkpoint blockade antibodies, or IFN-I treatment (192). These data suggest that CIS represents a promising target in immunotherapy especially in combination with other immunomodulatory agents (185).

### SOCS1

SOCS1 negatively regulates signaling of IFNs and IL-12 (193, 194) and plays an important role in DC and T cells suppressing antigen-presentation and antitumor immunity (195). *Socs1*^−/−^ mice die shortly after birth due to severe inflammation and uncontrolled IFN-γ signaling (196). *Socs1*^−/−^*Ifng*^−/−^ double-knockout mice survive until adulthood (196) and IL-12-treated NK cells isolated from these mice display an enhanced capacity to lyse YAC-1 target cells (197). However, *Socs1*^−/−^*Ifng*^−/−^ mice seem to have slightly reduced NK cell numbers in the periphery and hampered NK cell proliferation in response to IL-15 (57). A detailed analysis of the role of SOCS1 in NK cell development, maturation and function is pending.

### SOCS2

SOCS2 is closely related to CIS and induced by STAT5-activating cytokines, such as GH and IL-15 in mouse and human NK cells (57, 186, 187). SOCS2 represses NK cell development, as *Socs2*^−/−^ mice have increased NK cell numbers in bone marrow and spleen while T-, B- and myeloid cell numbers are unaltered (48). The increased NK cell numbers translate into enhanced tumor surveillance. In contrast to the situation in *Cish*-deficient mice and against the expectations, *Socs2*-deficiency does not enhance the cytotoxicity or IFN-γ production of NK cells. Intriguingly, the absence of SOCS2 boosts IL-15-induced JAK2/STAT5 activation in NK cells (48), which has commonly been believed to signal via JAK1 and JAK3.

In contrast to murine NK cells, knockdown of *SOCS2* has no impact on the IL-15-induced *in vitro* differentiation of primary human NK cell precursors, but severely diminishes the cytotoxic function of primary NK cells and the human NK cell line NK-92. The reduced cytotoxicity was assigned to impaired degradation and accumulation of the focal adhesion kinase PYK2 (198), which is involved in the formation of the NK/target cell synapse upon killing (199). Although it is clear that SOCS2 interferes with NK cell functions, the distinct roles of SOCS2 in human and mouse NK cells remain enigmatic.

### SOCS3

*Socs3*^−/−^ mice are embryonically lethal due to placental defects (200) and impaired fetal liver hematopoiesis (201). SOCS3 counteracts inflammation by inhibiting a variety of pro-inflammatory signaling pathways (202). Most prominently, SOCS3 inhibits gp130 receptor/STAT3 signaling by direct inhibition and/or ubiquitin-mediated degradation of the receptors or their associated JAK kinases (downstream of IL-6, IL-11, IL-27, OSM and LIF). SOCS3 was also shown to negatively regulate IL-12-induced STAT4 activation by blocking the IL-12Rβ2 subunit via its SH2 domain (203).

In mouse NK cells, SOCS3 is induced upon IL-15 signaling (57) and is a direct target gene of the helix-loop-helix protein ID2 (204). *Id2* deletion in NKp46^+^ cells leads to a complete absence of peripheral NK cells due to impaired IL-15 signaling. NK cell numbers are rescued by additionally deleting *Socs3* (204). However, loss of *Socs3* alone in the presence of ID2 does not alter the development, maturation or IL-15-induced proliferation of mouse NK cells (57, 204). CRISPR/Cas9-mediated disruption of *SOCS3* in human NK cells promotes proliferation and cytotoxicity (205), thereby suggesting that SOCS3 may be a useful target for NK cell-based immunotherapy. However, taking into account the detrimental effect of *Socs3*-deficiency in mice, any envisaged inhibitor treatment will have to be applied specifically on NK cells to avoid generic adverse side-effects.

## Concluding Remarks

The JAK/STAT pathway is evolutionary highly conserved and transmits extracellular signals to the nucleus modulating target gene transcription. Members of this pathway are frequently altered in cancer including malignancies of innate lymphocytes, making them an attractive target for drug development. Several JAK inhibitors are already used for the treatment of rheumatoid arthritis, psoriasis and myelofibrosis, and entered phase 2 and 3 clinical trials for the treatment of other inflammatory diseases and cancer. While the first clinically used inhibitors such as Ruxolitinib or Tofacitinib, proved to target multiple JAK kinases, more specific compounds found their way into clinical trials (206, 207).

We and others have previously shown that treatment with the JAK1/2 inhibitor Ruxolitinib substantially impairs NK cell functions leading to increased susceptibility to viral infections and tumor metastasis (49, 208). In line, in a mouse B cell lymphoma model Ruxolitinib treatment promotes tumor progression by enhancing NK cell-derived VEGF-A expression (122). On the other hand, Ruxolitinib treatment significantly reduces disease burden in the context of CD56^+^ T-cell large granular lymphocytic (T-LGL) leukemia (170) and restores impaired NK cell functions in patients harboring *STAT1 GOF* mutations (96).

JAK inhibitor treatment shall be carefully evaluated to identify the complex interplay and potential opposing effects on target and immune cells. While in the context of inflammatory and immune-related diseases JAK inhibitor-induced dampening of NK cell functions may be advantageous, NK cell malfunction in metastatic cancers should be precluded. Besides blocking JAK kinases, considerable effort is undertaken to develop specific STAT inhibitors. This could be of particular interest for the field of immunotherapy, as treatment with STAT3 or STAT6 inhibitors may enhance NK cell cytotoxicity. Finding ways to efficiently improve NK cell functions will promote the use of adoptively transferred NK cells in everyday clinics.

Targeting the negative regulators of the JAK/STAT pathway also holds great promise as novel immunotherapeutic strategy. In particular, CIS was shown to be a checkpoint in NK cell-mediated tumor control making it an attractive candidate for anti-tumor therapy. The expanding knowledge of immune checkpoints and potential drug candidates opens a new avenue for immunotherapy, yet the next challenge is to develop specific and stable compounds suitable for clinical use.

## Author Contributions

JT generated the figures. DG, VS, and EP drafted the figures and wrote the manuscript.

### Conflict of Interest

The authors declare that the research was conducted in the absence of any commercial or financial relationships that could be construed as a potential conflict of interest.

## Abbreviations

BRAF: rapidly accelerated fibrosarcoma isoform B
CHILP: common helper-like innate lymphoid precursor
CIS: cytokine inducible SH2-containing protein
*Cish*: gene coding for CIS protein
CLP: common lymphoid progenitor
CLPD: chronic lymphoproliferative disorder
DC: dendritic cell
DNAM1: DNAX accessory molecule 1 (CD226)
EILP: early innate lymphoid cell progenitor
EOMES: Eomesodermin
γc: common gamma chain
GATA3: GATA-binding protein 3
GH: growth hormone
GOF: gain-of-function
HSC: hematopoietic stem cell
HSV-1: herpes simplex virus 1
ID2: inhibitor of DNA binding 2
IFN: interferon
IFN-I: type 1 interferon [e.g., IFN-α (alpha), IFN-β (beta)]
IFN-II: type 2 interferon [IFN-γ (gamma)]
IFN-III: type 3 interferon [IFN-λ (lambda)]
IFNAR: interferon-α/β receptor
IL: interleukin
ILC: innate lymphoid cell
ILCP: innate lymphoid cell precursor
IRF9: interferon regulatory factor 9
ISGF3: interferon-stimulated gene factor 3
JAK: Janus kinase
*Klrk1*: gene coding for NKG2D protein
LCMV: lymphocytic choriomeningitis virus
LIF: leukemia inhibitory factor
LOF: loss-of-function
mAB: monoclonal antibody
MCMV: mouse cytomegalovirus
MEK: mitogen-activated protein kinase kinase
MHC: major histocompatibility complex
NK: natural killer
NKG2D: natural killer receptor group 2, member D
NKP: NK lineage-restricted progenitor
NKTCL: NK/T-cell lymphoma
OSM: oncostatin M
PMA: phorbol 12-myristate 13-acetate
PYK2: protein tyrosine kinase 2
SCID: severe combined immunodeficiency
SH2: Src homology 2
SOCS: suppressor of cytokine signaling
STAT: signal transducer and activator of transcription
T-BET: T-box expressed in T cells
*Tbx21*: gene coding for T-BET
TGF-β: transforming growth factor beta
Th1: T-helper cell type 1
Th2: T-helper cell type 2
T-LGL: T-cell large granular lymphocytic
TRAIL: tumor necrosis factor-related apoptosis-inducing ligand
TYK2: tyrosine kinase 2
RORγt: retinoic acid-related orphan receptor gamma t
U-STAT: unphosphorylated STAT
VEGF-A: vascular endothelial growth factor A.
